# *YES, WE COPE*: Dyadic Coping as a Mediator Between Perceived Relationship Quality and Emotional Representation of COVID-19

**DOI:** 10.1007/s10608-023-10379-4

**Published:** 2023-05-04

**Authors:** Ana Paula Relvas, Laura Lacomba-Trejo, Alda Portugal, Claudia Chiarolanza, Sofia Major, Catarina Rosa, Luciana Sotero, Ashley K. Randall

**Affiliations:** 1 Present Address: Center for Social Studies, University of Coimbra & Faculty of Psychology and Education Sciences, University of Coimbra, Coimbra, 3000-115 Portugal; 2 Department of Social Psychology, Faculty of Psychology and Speech Therapy, Universitat de València, Av. Blasco Ibáñez, 21. 46010, Valencia, Spain; 3 Center of Social Studies, University of Coimbra, 3000-115 Coimbra & Department of Psychology, University of Madeira, 9020-105, Funchal, Portugal; 4 Department of Dynamic and Clinical Psychology, and Health Studies, Sapienza University of Rome, Piazzale Aldo Moro, 5, Rome, 00185 Italy; 5 Faculty of Social and Human Sciences, University of the Azores, Center for Research in Neuropsychology and Cognitive Behavioral Intervention, University of Coimbra, Coimbra, Portugal; 6 Department of Education and Psychology, University of Aveiro, Aveiro, Portugal; 7 Honors Faculty, Senior Global Futures Scientist Counseling and Counseling Psychology, Arizona State University, 446 Payne Hall Tempe, Arizona, 85281 AZ USA; 8 Center of Social Studies, University of Coimbra & Faculty of Psychology and Education Sciences, University of Coimbra, Coimbra, 3000-115 Portugal

**Keywords:** COVID-19, dyadic coping, perceived quality of relationships, couple, emotional response

## Abstract

**Background:**

The COVID-19 pandemic has brought about social changes that have impacted the functioning and dynamics of couples in a romantic relationship, arising from the overwhelming amount of added stress they have endured. Specifically, the divorce rate in Portugal has increased after lockdown, which underscores the adverse consequences of COVID-19 on couples. A lower quality of the couple’s relationship may worsen the emotional representation of COVID-19; however, the literature suggests that perceived partner dyadic coping responses have a great influence on adverse events. The aim of the present study was to assess the mediating role of partner coping in the association between relationship quality and emotional representation of COVID-19. We also sought to observe whether the length of the relationship moderates this association.

**Methods:**

Participants (*N* = 528) were adults living in Portugal (84.7% female) currently in a romantic relationship with their current partner for at least 1 year. Online data collection.

**Results:**

We found that relationship quality predicted COVID-19 emotional representation, but this association was fully mediated by total dyadic coping. This association was especially significant in couples with a shorter length of time in the relationship.

**Conclusions:**

We point out the importance of dyadic coping as a protective factor against emotional distress to cope with the ongoing stressors associated with the COVID-19 pandemic. These data suggest the need for relationship education programs that promote positive coping between partners.

**Supplementary Information:**

The online version contains supplementary material available at 10.1007/s10608-023-10379-4.

## Introduction

New and significant challenges created by the COVID-19 pandemic has led to profound changes in the way we have been living across the globe since the beginning of 2020. Although Portugal was the last country in Europe to be afflicted by COVID-19, the “state of alarm” was nevertheless declared on 15 March 2020. Between April and May 2020 1,410 people had died, and 30,622 people had been infected, significantly less than in other European countries (Johns Hopkins University of Medicine, 2021). The state of alert in Portugal called for general containment measures, capacity limits in public venues, and teleworking recommendations, among others (República Portuguesa XXII Governo, 2022). The prevention measures implemented were social distancing and home confinement (including restrictions on circulation outdoors, and the need to work from home or home-school children). For these reasons, individuals, couples and families radically changed their daily routines, especially during the first wave of COVID-19 when little was known about how the illness would progress (Mertens et al., 2020). In this sense, one of the challenges of the pandemic emerged when couples were required to spend extended amounts of time together, isolated at home for weeks on end, conditions which had an impact on intimate relationships (Williamson, 2020). According to data provided by Justice Ministry to Portuguese social media (Faria, 2021), fewer divorces and marriages occurred during lockdown; however, divorce rates increased after mandatory lockdown.

In this changing and stressful context for individuals, couples and families, the emotional representation of illness emerged as an important variable. In fact, along with the cognitive representation, emotional representation is one of the two components of illness representation considered in Leventhal et al. (1984) model as pointed out by Broadbent et al. (2006). It encompasses negative reactions such as fear, anger and distress and is of paramount importance because it correlates strongly with the ignorance and lack of knowledge about the illness. The literature has also pointed out that the person’s emotional (and cognitive) representation of the illness is highly relevant in predicting emotional adjustment to it (see, for instance, the meta-analysis review Hagger & Orbell, 2003). Besides, some studies show that people who reported “a heightened fear response to an unknown health threat” reveal “a highest emotional” representation of the illness (Broadbent et al., 2006, p. 636). Emotional representation is therefore strongly relevant in highly health threatening and unknown illnesses, such as COVID-19. The health threat of COVID-19 was particularly associated with higher risk populations, such as the elderly and children (Eiguren et al., 2021; Idoiaga et al., 2020). Nevertheless, COVID’s threat impacted individually and in couples’ relationships (Relvas et al., 2020) implying a major stress and requiring effective coping measures for a good adjustment. According to the Family Adjustment and Adaptation Response Model (FAAR Model; McCubbin & Patterson 1983; Patterson, 1988) these adjustment/adaptation processes are mediated by family “meanings”, that is, the beliefs and representations about the stressor, in this case about the COVID-19 disease.

Studies reported that it is vitally important to understand how partners perceive the disease, as it may or may not help them adjust emotionally to the pandemic (Stephenson et al., 2020; Walsh & Stephenson, 2021). It has been observed that couples who had a longer lasting relationship showed a COVID-19 greater impact and less satisfaction with their partner (Mousavi, 2020). So, it seems that in intimate relationships, the partner plays a fundamental role in this balancing game between stress and the adjustment/adaptation movements. We know that it was proved that the experience of stress outside the relationship (i.e., external stressors such as work, financial issues, illness) can spill over into the relationship, thus generating stress between and having significant and negative impact on perceived quality of relationships (Chua et al., 2021; Conger et al., 1999; Cutrona et al., 2018; Karademas & Roussi, 2017). From this point of view, it is important to note that the COVID-19 pandemic has heightened socio-economic, political and health concerns and stress, that is to say an extreme emotional representation of the illness (Donato et al., 2021; Mertens et al., 2020). By the other side, COVID’s impact has decreased partner-related protective factors such as support, affection and positive climate (Fraenkel & Cho, 2020). Briefly, we can say that the pandemic has increased the risk factors for stress and reduced the protective ones both in individuals and couples. Recent meta-analysis among the general population concluded that COVID-19 not only causes physical health concerns but also results in a number of psychological disorders, namely impacts on the prevalence of individual anxiety, depression and psychological stress (e.g., Jin et al., 2021; Salari et al., 2020).

The Systemic-Transactional Model of Dyadic Coping acknowledges that partners’ experiences of stress and coping are interdependent (Bodenmann, 1995; Bodenmann et al., 2019). Bodenmann (1995) defines dyadic coping as a process partners use to cope with stressors, taking into account both individual and joint strategies for coping with stress. Indeed, diverse studies (Falconier et al., 2015; Karademas & Roussi, 2017; Vedes et al., 2016; Xu et al., 2016; Randall & Messerschmitt-Coen, 2019) suggest that perceived partner positive dyadic coping strongly predicts perceived quality of relationship, contributing to reducing stress. Concerns about COVID-19 may tend to lessen the sense of well-being, but if explicit stress communication and partner coping are increased, well-being is not affected (Donato et al., 2021). In this sense, the way in which the partner responds to the other’s COVID-19- related stressors are said to protect individuals from the negative effects of pandemic stressors (Balzarini et al., 2023; Donato et al., 2021). Briefly, positive dyadic coping is commonly associated with decreased reports of stress and higher levels of satisfaction and perceived quality of relationships (Bar-Kalifa et al., 2022; Bodenmann et al., 2019; Chen et al., 2021; Cutrona et al., 2018; Donato et al., 2021; Harrison et al., 2021; Ogan et al., 2021; Vedes et al., 2016).

Understanding the factors that promote couples’ adjustment to stressful life events can help to generate intervention programmes that improve this adjustment. Given this, it is of paramount importance to examine how couples were coping with perceived impact of COVID-19. Considering The theoretical framework, to achieve that goal it seems important to assess the influence of relational quality on COVID-19 threat perception. As far as we know, these aspects have not yet been fully evaluated in Portuguese couples.

### Objectives of the Current Study

There is little doubt as to the tremendous impact the COVID-19 pandemic has had on individuals’ and couple’s well-being (Canet-Juric et al., 2020; Donato et al., 2021; Goodboy et al., 2021; Lillie et al., 2021). It is necessary to understand how perceived impact of COVID-19 may be associated with individuals’ perceived relationship quality, when mediated by dyadic coping (Goodboy et al., 2021; Jörngården et al., 2006). To our knowledge, these aspects have not yet been studied in Portuguese couples. Furthermore, previous research compared results from couples from different countries, but does not analysed data from each country independently (Randall et al., 2022). Given the significance of a deeper understanding of the processes regarding the pandemic stressors on couple relationships and the importance of dyadic coping, it seems highly relevant to analyse the association between these variables during a critical period of pandemic. Doing so will help to develop psychological interventions that improve dyadic coping and thus the perception of the quality of the couple’s relationship. The first objective of the present research (O1) is to study the interplay between dyadic coping and relationship quality and its effect on the emotional representation of COVID-19. We hypothesise, on the one hand, (O1-H1) that the perceived relationship quality will be negatively associated with the emotional representation of COVID-19; on the other hand, (O1-H2) dyadic coping will be negatively associated with the emotional representation of COVID-19. Finally, (O1-H3) the perceived relationship quality will be positively associated with dyadic coping.

Regarding the second objective, (O2) we will examine whether one of the variables (coping dyadic or relationship quality) is more important than the other in predicting the emotional representation of COVID-19. Finally, we will investigate whether the length of relationship moderates this interplay (O3). We hypothesize (O3-H1) that the relationship length moderates the interplay between dyadic coping and perceived relationship quality in predicting the emotional representation of COVID-19.

## Method

### Participants

The following inclusion criteria were used: being at least 18 years of age, living in Portugal, and being in a romantic relationship for at least 1 year and living with their partner. Participants took part in an international study (“COVID-19: Transcultural study on Global Stressors Effects on Couples”) conducted across 27 countries (the study was pre-registered at the following link: https://osf.io/s7j52) (Randall et al., 2022). For the present study, only data from Portugal is presented. From the 1,310 people who had access to the survey, 737 were eligible according to the inclusion criteria. Of those, 198 were excluded because they did not respond to all the protocol’s questionnaires and 11 because they took more than 24 h to complete the survey (this criterion was assumed by the international team as indicating a possible bias).

A total of 528 participants were included in the present study. Most of them were female (*n* = 447; 84.70%), aged between 19 and 70 years-old (*M* = 39.45; *SD* = 9.99). Participants were highly educated, as most of them had a higher education degree (*n* = 429; 81.30%) and were employed (*n* = 421; 79.70%) at the time data was collected. More than half of the participants were married (*n* = 270; 51.10%) and had children (*n* = 297; 56.30%). The length of the relationship of the partners ranged from 1 to 53 years (*M* = 16.31; *SD* = 10.63). Table 1 presents the socio-demographics of the participants and the characteristics of the couples.

**Table 1:** *Participants’ Sociodemographic and Couple’s Characteristics*

Characteristic	*n* (*N* = 528)	%
Sex		
Male	80	15.20
Female	447	84.70
Other	1	0.20
Age^a^		
18–29	91	17.40
30–39	185	35.40
40–49	155	29.60
50–59	73	14.00
≥ 60	19	3.60
Education		
≤ 4 years	2	0.40
6 years	6	1.10
9 years	13	2.50
12 years/Professional	78	14.80
Graduate degree	429	81.30
Occupational status		
Employed	421	79.70
Unemployed	69	13.10
Other situation	38	7.20
Marital status		
Committed relationship	87	16.50
Engaged	171	32.40
Married	270	51.10
Having children		
Yes	297	56.30
No	231	43.80
Person had suffered COVID-19		
Yes	4	0.76
No	481	91.10
Not sure	43	8.14
Partner had COVID-19		
Yes	1	0.20
No	491	93.00
Not sure	36	6.80
Family member had experienced COVID-19		
Yes	41	7.74
No	458	86.47
Not sure	29	5.22

^a^Age was not available for five participants

### Measures

**The Perceived Relationship Quality Component Inventory (PRQC)**. Relationship quality was measured using a reduced perceived quality of relationships version of the PRQC (Fletcher et al., 2000; Portuguese version: Costa & Brody 2007). The PRQC consists of six items based on a 7-point Likert scale from 1 “*Not at all*” to 7 “*Extremely*” (e.g., “How much do you trust your partner?”). The score of the questionnaire is obtained by summing up the items. Considering the importance of the PRQC for this study and given that this scale has only a Portuguese translation without a validation study, the psychometric characteristics of this study sample (*N* = 528) – validity and reliability – are presented. A Mardia’s test (Mardia, 1970) revealed that data are not multivariate normal, *g1p* = 21.80, *χ*_*Skew*_ = 1999.06, *p* < .001; *g*^*2*^*p* = 102.73, *Z*_*Kurtosis*_ = 65.56, *p* < .001; χ_SMSkew_ = 2013.07, *p* < .001. Given the ordinal nature of data, a confirmatory factor analysis using Weighted Least Squares with Mean and Variance adjustment (WLSMV) (Finney & DiStefano, 2006) as estimator was performed. This one-factor model revealed a good global adjustment (CFI = 1.00, TLI = 1.00, SRMR = 0.021, RMSEA < 0.01, RMSEA 90% CI [0.000; 0.001]), all items reached high factor weights (*λ* ≥ 0.50 – See Supplementary Material, Figure S1) and appropriate individual reliabilities (*R*^*2*^ ≥ 0.25), showing good local adjustment. This result indicates that all items are a reflection of the latent factor being measured (Hair et al., 2010).

To evaluate whether the items had good latent trait discrimination, based on recommendations from Muraki (1997), a Polytomous Item Response Theory analysis using generalized partial credit model was performed (See Supplementary Material, Table S1). Considering the discrimination parameter (a), all items reached discrimination values above the acceptable (≥ 0.70) (Embretson & Reise, 2000), guaranteeing greater confidence in the measure.

Regarding reliability, ordinal Cronbach’s α based in polychoric matrix reached the value of 0.96, revealing very good internal consistency (Nunnally & Bernstein, 1994). The construct of perceived relationship quality was psychometrically validated by results of factorial validity and reliability obtained for one-factor solution in our sample, giving it robustness for later analyses.

**The Brief Illness Perception Questionnaire (Brief IPQ)**. The Brief IPQ (Broadbent et al., 2006; Portuguese version: Araújo-Soares et al., n.d) was used to assess the perceived threat of illness. It consists of 8 items with a Likert-type response from 0 to 10 (minimum agreement to maximum agreement). It has a ninth open-ended item that takes into account the causes of the illness. Each of the items can be used separately (consequences, duration, personal control, treatment control, symptoms, worry, emotional response and understanding of the illness) or as a total illness threat score (Broadbent et al., 2006; Valero-Moreno et al., 2020). In the present study, Item 8, that’s it’s to say emotional response/ representation of COVID-19, was used [*To what extent has the COVID-19 pandemic affected you emotionally (e.g., made you angry, scared, upset or depressed)?*]. This item is answered from 0 to 10, with 0 being “*Not at all emotionally affected*” and 10 being “*Extremely emotionally affected*”. It was decided to use the emotional representation item because of its relevance to periods of crisis, which involve emotional activation to cope with stress. Previous studies show adequate psychometric properties (Broadbent et al., 2006; Valero-Moreno et al., 2020).

**The Dyadic Coping Inventory (DCI)**. The DCI was used to assess perceptions of partners’ dyadic coping while experiencing stress (Bodenmann, 2008; Portuguese version: Vedes et al., 2013). The DCI consists of 37 items rated on a 5-point Likert scale ranging from 1 “*Very rarely*” to 5 “*Very often*”. The scale has five subscales, and a total score (Total Dyadic Coping - TDC), which was used in the present study. The TDC was calculated through the mean on 35 items (recoding items 7, 10, 11, 15, 22, 25, 26, 27 and excluding the item 36 and 37 since these items evaluate how satisfied individuals are with their DC, though are not used to describe DC behaviour itself). Previous literature has indicated that the scale has adequate psychometric properties (Bodenmann, 2008; Vedes et al., 2013; Xu et al., 2016). The Ordinal Cronbach’s α of our sample/TDC (*N* = 528) achieved the value of 0.95, showing an excellent internal reliability.

### Procedure

Participants were recruited through institutional websites (e.g., participant universities) and social media sites (e.g., Facebook) and directed to an online survey link to participate in the study. Data -was collected online via Qualtrics during the first lockdown in Portugal (April-May 2020). e-Consent was obtained from all participants before completing the questionnaires. Five screening questions were used to determine participant eligibility (inclusion criteria). The study was approved by the Ethics Committee of the Faculty of Psychology and Educational Sciences of the University of Coimbra.

### Analytic Outline and Statistical Analysis

Considering the research objectives and hypotheses, we first carried out descriptive statistics and Pearson correlations with the SPSS v26 programme (O1-H1, H2, H3). Then, taking into account the aim of the study, a moderated mediation analysis was performed using R (CRAN PROJECT) (R core team, 2021). The mediation analysis was carried out to answer O2, and the mediated moderation analysis was intended to verify whether the previously evaluated mediation was moderated by the relationship time (O3-H1).

## Results

Table 2 includes descriptive statistics for relationship satisfaction and dyadic coping in the sample. Moderate levels of threat perception (emotional representation) and high levels of dyadic coping and partner satisfaction are observed. To test O1 - H1, H2 and H3, we carried out Pearson correlations (see Table 2). We found that perceived relationship quality was positively and linearly associated with dyadic coping (*r*_*x*_ = 0.71, *p* < .0001), and negatively and linearly associated with COVID-19 emotional representation (*r*_*x*_ = − 0.12, *p* = .006) and with relationship time (*r*_*x*_ = − 0.09, *p* = .048). Dyadic coping was negatively related to the emotional representation of COVID-19 (*r*_*x*_ = − 0.18, *p* < .0001).

**Table 2:** *Descriptive Statistics and Correlations*

	*M*	*SD*	*P25*	*P50*	*P75*
PRQC	36.54	6.92	34.00	38.00	42.00
DCI	3.83	0.62	3.45	3.86	4.28
IPQ-E	5.88	2.45	4.00	6.00	8.00
REL-T	16.31	10.63	8.00	14.00	23.00
	1	2	3	4	
1. PRQC	1				
2. DCI	0.71***	1			
3. IPQ-E	− 0.12**	− 0.18***	1		
4. REL-T	− 0.10*	− 0.16**	0.07	1	

*Note*. PRQC = Perceived Relationship Quality Component Inventory; DCI = Dyadic Coping Inventory (Total Dyadic Coping); IPQ-E = Brief Illness Perception Questionnaire - Emotional Representation COVID; REL-T = Relationship Time; *p < .05 ** *p* < .01 ***p < .001

To test O1-H1, H2, H3 and O2, a mediation analysis was performed wherein we examined whether dyadic coping mediated the association between perceived relationship quality and emotional representation of COVID-19 as measured by item 8 of the Brief IPQ (perceived illness threat questionnaire) (Table 3). The results showed that perceived relationship quality is negatively associated with emotional representation of COVID-19 (O1-H1). Indeed, the total effect of PRQC on Brief IPQ (c + a × b) was significant (*β* = -0.417, *z* = -2.732, *p* = .006). Analysing the indirect effects, results revealed that dyadic coping significantly mediated the association between perceived relationship quality and emotional representation of COVID-19, *ab* = − 0.045, z = -2.959, *p* = .003, *95% CI* [-0.08, -0.02)]. Specifically, results showed that relationship quality was positively associated with dyadic coping (O1-H3), which was in turn negatively associated with emotional representation of COVID-19 (O1-H2; see Table 3). However, the results also suggested that without the indirect effect, the association between the perceived relationship quality and the emotional representation of COVID-19 would be non-significant (direct effect: *β* = -0.004, *z* = 0.167, *p* = .867). Indeed, the dyadic coping accounts for 92.67% of the relation between the perceived relationship quality and the emotional representation of COVID-19 (O1).

**Table 3:** *Path Estimates of Mediation Analysis*

							95% Confidence Interval			
			Label	Estimate	*SE*	Lower		Upper	*Z*	*p*
Perceived Relationship Quality	→	Dyadic Coping	a	0.06	0.00	0.06		0.07	21.89	< .001
Dyadic Coping	→	Emotional Representation COVID	b	-0.92	0.24	-1.39		-0.42	-3.82	< .001
Perceived Relationship Quality	→	Emotional Representation COVID	c	0.02	0.02	-0.03		0.06	0.79	.432

**Fig. 1:**
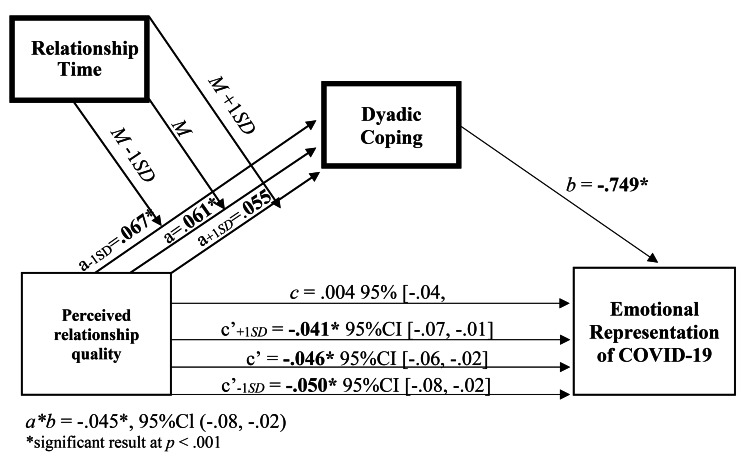
*Path Diagram of Tested Moderated Mediation Model*

In order to assess whether the previous mediation effect of dyadic coping was moderated by relationship length (O3-H1), a moderated mediation analysis was conducted. Results showed that there was no association between relationship length and dyadic coping (*β* = 0.001, SE = 0.007, *t* = 1.79, *p* = .074). However, the interaction between perceived relationship quality and relationship length was statistically significant, (*β*= -0.001, SE = 0.001, *t* =- 2.60, *p* = .01), suggesting that relationship length moderated the association between perceived relationship quality and dyadic coping. As shown in Fig. 1, simple slopes analysis revealed that the mediation effect of dyadic coping between perceived relationship quality and emotional representation of COVID is stronger for couples with less time in a relationship than for couples in a longer relationship. This difference was statistically significant (95% *CI* [0.001, 0.025]). This result shows that as younger couples have a greater positive relationship between relationship quality and dyadic coping and as this is negatively related to emotional representation of COVID, younger couples are more dependent on good dyadic coping to manage the emotional representation of COVID.

## Discussion

This study was conducted to assess the interplay between dyadic coping and relationship quality and its effect on the emotional representation of COVID-19. Similarly, we wanted to observe which variable (dyadic coping or relationship quality) was the most relevant for the prediction of the COVID-19 emotional response. Finally, we wanted to evaluate the role of the relationship length.

For (O1, H1) we found that higher relationship quality was related to a less threatening representation of COVID-19. These findings confirm previous studies relating relationship quality to illness perception (Randall et al., 2022). Regarding dyadic coping (O1, H2) we found that it was associated with lower perceived threat of COVID-19. This seems to suggest that dyadic coping strategies could be a protective factor for perceived relationship quality during the pandemic. With reference to (O1, H3), we observe that the quality of the intimate partner relationship is associated with dyadic coping. Previous literature had pointed out the association between the quality of the couple’s relationship and the couple’s ability to cope with adversity (Pieh et al., 2020). These results support the assumption of the Systemic-Transactional Model of Dyadic Coping (Bodenmann, 1995). Thus, the better the couple’s stress coping strategies, the lower the impact of stressful events on the perceived quality of the couple’s relationship (Bodenmann, 1995).

Regarding (O2), we observed that dyadic coping was the most relevant variable for predicting the emotional perception of COVID-19. Previous work points to the importance of dyadic coping in dealing with adversity (Bodenmann, 1995). However, these results are particularly novel because although other studies have recognised the relationship between the perceived quality of the couple’s relationship and stress, in our work this association is mostly explained by the couple’s dyadic coping. In fact, this association only exists due to the indirect effect, i.e., the perceived relationship quality is positively associated with dyadic coping and dyadic coping, in turn, negatively affects the emotional representation of COVID-19.

The previous literature had identified the role of dyadic couple coping in situations of physical and mental illness (Chen et al., 2021; Karademas, 2022; Meier et al., 2021). However, the mediating effect of this variable in the context of COVID-19 was not known. In this sense, the concept of “we-illness” applies to actual circumstances of physical or mental illness in one of the partners. Thus, couples deal with illness as a shared event, rather than as an individual experience (Falconier et al., 2015). Although in our sample most people had not had contact with COVID-19 (91.1%), they were confronted with an event that caused great uncertainty and a sense of threat, in this experience of an unprecedented social situation.

Finally, as for (O3, H1), we observe that younger couples are more dependent on good dyadic coping to manage COVID-19 emotional representation. Moreover, this study allows us to conclude that the relationship length moderates the association between the perceived quality of the couple’s relationship and dyadic coping. When couples have been together for a shorter period of time, this association was stronger. Consequently, the mediation effect of dyadic coping between perceived relationship quality and the emotional representation of COVID-19 is moderated by the relationship length. In the same way, this mediation role was stronger for couples who have been together for a shorter period. This result seems to indicate that dyadic coping plays a central role for the perception of relationship quality in young couples, but a less important role in couples in more long-term relationships. This is a very relevant finding considering the lack of clues in the literature about relationship length and dyadic coping. Someway our results are in line with the findings reported by Landis et al. (2013), showing that for older couples the individual support perception was more important for marital satisfaction than dyadic coping. In fact, most of the studies between dyadic coping and relationship satisfaction correlation were made including samples of young and middle-aged married individual (Bodenmann, 2000) and the predictive power of dyadic coping for older adults’ relationship satisfaction has received relatively little attention. According to literature, older couples showed a reduced conflict potential, more sources of pleasure and are experts at regulating their own and their socials partners’ emotions (Fingerman & Charles, 2010; Levenson et al., 1993). So, in individuals in older romantic relationships maybe there are other important variables that along with the dyadic coping would better explain the relationship satisfaction, assuming the dyadic coping a more determinant role for individuals in a young romantic relationship. Regarding these findings, psychological interventions targeting dyadic coping would be useful to prevent less adaptive emotional representations of COVID-19 and similar stressful events in the future, mainly in young couples.

### Limitations, strengths and future lines

Nevertheless, despite the potential of our study, the characteristics of our sample make it difficult to generalise the results. In particular, more than 80% of the participants were female and they had a high level of education. However, most research conducted during the first wave shows the same limitation. In addition to the above, the stringent inclusion criteria, namely requiring participants to have been in the relationship for at least one year and living together, limit the generalisability of the results. In addition, the sample was collected online, as this was the only way possible during confinement. This evaluation format may have biases which in future studies should be controlled at least with an infrequency scale. Besides there are some relevant variables which were not evaluated (e.g., cohabitation) that might highlight our results in a most interesting way, what may also be seen as a study’s limitation and so considered in future studies. Further studies are needed to replicate this research, perhaps through the collection a probability sample that more adequately represents the characteristics and particular features of Portuguese couples during the COVID-19 pandemic. Likewise, future studies should consider how dyadic coping influences other factors in the IPQ (such as personal control of illness, control of treatment, symptoms, worry, emotional response and understanding of illness). Similarly, the role of time spent together in these partnerships should be explored (e.g. study the relationship length as a moderating factor between dyadic coping and relationship satisfaction), as well as the specific time that the couples were cohabiting. Finally, it would be interesting for future research to conduct longitudinal studies and take into account the whole dyad. However, longitudinal studies may not be well-suited to times of pandemics, due to the broad societal changes that occur within a short period of time.

Despite the limitations, our study has great potential. It is the first study conducted in Portugal during the COVID-19 pandemic, which evaluates the association between the perceived relationship quality and the emotional representations of COVID-19, including couple coping as a mediating element, and assessing the impact of the time spent in the relationship. The findings of our study point to the importance of couple coping strengths, which is a key aspect of adjustment to stressful life situations. Psychological intervention programmes should strengthen positive couple coping, as this protective factor can help in the current situation and in future pandemics. Thus, our results are particularly relevant for future prevention. They are applicable to the next waves of infection, but also to possible future pandemics. Promoting dyadic coping through psychotherapeutic interventions can improve the mental health of couples, which in turn can have a holistic impact on their well-being.

Future research should focus on probabilistic, longitudinal and randomised studies. In this way, more generalizable results may be obtained. However, it has been pointed out that given the rapidly changing nature of the pandemic, longitudinal studies may not be appropriate. On the other hand, other variables such as the possible death of a close family member during the pandemic, as well as other risk and protective factors for adversity such as family structure, could be controlled for. Therefore, we note that it is important to recognise how psychosocial, cultural, political and health factors may have influenced participants’ perception of stress, coping skills and the quality of the couple’s relationship.

## Conclusions

Overall, our results show that dyadic coping is a highly relevant variable for the prediction of COVID-19 emotional representation, much more so than partner satisfaction. This is especially relevant for young couples, who may be more vulnerable to stress than their older counterparts. We conclude that the present work can form a basis for future studies where interventions focusing on dyadic coping are developed with the aim of improving couples’ well-being in the face of adversity.

Tables and Figures.

## Electronic Supplementary Material

Below is the link to the electronic supplementary material.

Supplementary Material 1

## Data Availability

*(data transparency)*: The datasets generated and/or analyzed during the current study are available from the corresponding author upon reasonable request.

## Code Availability

*(software application or custom code)*: Not applicable.
