# Supplementary material for: YES, WE COPE: Dyadic Coping as a Mediator Between Perceived Relationship Quality and Emotional Representation of COVID-19
Source: Cognit Ther Res. 2023 May 4:1–11. Online ahead of print. doi: 10.1007/s10608-023-10379-4 (PMC10156576; doi:10.1007/s10608-023-10379-4)
Supplement: Supplementary file 1 — Supplementary Material 1 [file 10608_2023_10379_MOESM1_ESM.docx]

**Supplementary Material**

**Table S1**

*Polytomous Item Response Theory Parameters (PL2 model)*

| Item | a | b1 | b2 | b3 | b4 | b5 | b6 | b7 |
| --- | --- | --- | --- | --- | --- | --- | --- | --- |
| PRQC1 | 4.55 | -2.62 | -2.28 | -1.70 | -1.50 | -1.07 | -0.53 | 0.42 |
| PRQC2 | 2.12 | -2.93 | -1.79 | -1.99 | -1.55 | -1.54 | -1.40 | -0.56 |
| PRQC3 | 3.43 | -2.64 | -2.28 | -1.90 | -1.43 | -1.27 | -0.65 | -0.04 |
| PRQC4 | 1.37 | -3.18 | -2.65 | -1.89 | -2.11 | -1.61 | -1.35 | -0.46 |
| PRQC5 | 2.565 | -2.26 | -2.00 | -1.72 | -1.44 | -0.98 | -0.51 | 0.07 |
| PRQC6 | 3.45 | -2.69 | -2.13 | -1.91 | -1.66 | -1.36 | -1.01 | -0.41 |

**Figure S1**

*Confirmatory Factor Analysis Diagram with One-Factor Solution to PRQC.*

**PRQC**

**PRQC3**

**PRQC1**

0.89

0.73

0.91

0.81

0.92

0.86

**PRQC6**

**PRQC5**

**PRQC4**

**PRQC2**
